# Electroacupuncture suppresses neuronal ferroptosis to relieve chronic neuropathic pain

**DOI:** 10.1111/jcmm.18240

**Published:** 2024-03-20

**Authors:** Chunchun Xue, Wenyun Kui, Aiping Huang, Yanan Li, Lingxing Li, Zhen Gu, Lei Xie, Shuyi Kong, Jun Yu, Hongfeng Ruan, Kaiqiang Wang

**Affiliations:** ^1^ Department of Pain, Shanghai Municipal Hospital of Traditional Chinese Medicine Shanghai University of Traditional Chinese Medicine Shanghai China; ^2^ Institute of Orthopaedics and Traumatology The First Affiliated Hospital of Zhejiang Chinese Medical University (Zhejiang Provincial Hospital of Traditional Chinese Medicine) Hangzhou China

**Keywords:** electroacupuncture, ferroptosis, glutathione peroxidase 4, neuronal protection, neuropathic pain, oxidative stress

## Abstract

Growing evidence supports the analgesic efficacy of electroacupuncture (EA) in managing chronic neuropathic pain (NP) in both patients and NP models induced by peripheral nerve injury. However, the underlying mechanisms remain incompletely understood. Ferroptosis, a novel form of programmed cell death, has been found to be activated during NP development, while EA has shown potential in promoting neurological recovery following acute cerebral injury by targeting ferroptosis. In this study, to investigate the detailed mechanism underlying EA intervention on NP, male Sprague‐Dawley rats with chronic constriction injury (CCI)‐induced NP model received EA treatment at acupoints ST36 and GV20 for 14 days. Results demonstrated that EA effectively attenuated CCI‐induced pain hypersensitivity and mitigated neuron damage and loss in the spinal cord of NP rats. Moreover, EA reversed the oxidative stress‐mediated spinal ferroptosis phenotype by upregulating reduced expression of xCT, glutathione peroxidase 4 (GPX4), ferritin heavy chain (FTH1) and superoxide dismutase (SOD) levels, and downregulating increased expression of acyl‐CoA synthetase long‐chain family member 4 (ACSL4), malondialdehyde levels and iron overload. Furthermore, EA increased the immunofluorescence co‐staining of GPX4 in neurons cells of the spinal cord of CCI rats. Mechanistic analysis unveiled that the inhibition of antioxidant pathway of Nrf2 signalling via its specific inhibitor, ML385, significantly countered EA's protective effect against neuronal ferroptosis in NP rats while marginally diminishing its analgesic effect. These findings suggest that EA treatment at acupoints ST36 and GV20 may protect against NP by inhibiting neuronal ferroptosis in the spinal cord, partially through the activation of Nrf2 signalling.

## 1 INTRODUCTION

Neuropathic pain (NP) represents a significant clinical challenge, affecting up to 7% ~ 10% of the population worldwide.^1^ It has been proved that chronic NP suffering from allodynia and hyperalgesia lasts for a long period, which causes great damage to the physical and mental health of patients.^2^ Despite remarkable advancements in NP research, current pharmacological interventions still fall short in delivering complete pain relief due to the intricate nature and progression of NP.^3^ Therefore, there is a pressing need to find a reliable strategy to promote the treatment of NP.

Electroacupuncture (EA), renowned for its potent analgesic effects and minimal side effects, has been extensively employed in NP treatment.^4^, ^5^ A randomized clinical trial showed the efficacy of incorporating EA and biofeedback therapies with conventional treatment in chronic pain management.^6^ Preclinical studies have reported that EA can effectively ameliorate chronic pain syndromes in NP animal models^7^, ^8^, ^9^; however, the underlying mechanisms of EA's therapeutic effects on NP remain incompletely elucidated.

Ferroptosis, a novel type of cell death, is characterized by iron‐dependent accumulation of lethal lipid peroxides.^10^, ^11^, ^12^ Unlike other forms of cell death, ferroptosis exhibits distinct morphological, biochemical and genetic properties. Biologically, it is associated with iron overload, increased lipid peroxide production and elevated oxidative stress.^13^ Genetic hallmarks typically include downregulation of anti‐ferroptosis factors, such as xCT and glutathione peroxidase 4 (GPX4), and upregulation of pro‐ferroptosis markers like acyl‐CoA synthetase long‐chain family member 4 (ACSL4).^14^ Morphologically, ferroptotic cells are characterized by mitochondrial structural impairments, identified as reduced or vanished inner membrane cristae.^13^ Emerging evidence indicates that the involvement of ferroptosis in the progression of NP induced by chronic constriction injury (CCI),^15^ whereas inhibition of ferroptosis by ferrostatin‐1 (Fer‐1) has been found to rescue the CCI‐induced pain hypersensitivities.^16^ These findings imply that spinal ferroptosis contributes the NP progression, making the suppression of ferroptosis a potential target for NP treatment. Recent evidence suggests that EA may promote recovery of neurological functions in acute cerebral injury by inhibiting ferroptosis.^17^ Further findings from acute cerebral ischemia–reperfusion have also demonstrated that EA could suppress ferroptosis in brain tissue by regulating oxidative stress and iron metabolism‐related proteins.^18^ However, the precise impact of EA on the regulation of ferroptosis in NP treatment remains unknown.

In this study, we employed CCI‐induced NP models in rats to determine the potential of EA in ameliorating NP through inhibition of spinal cord ferroptosis. We will scrutinize the specific ferroptosis‐related biological indicators and further elucidate the cell types involved in ferroptosis modulation during EA treatment for NP progression. Moreover, we unveiled the defending ferroptosis regulatory system mediated by Kelch‐like ECH‐associated protein 1 (Keap1)—NF‐E2‐related factor 2 (Nrf2) signalling in the spinal cord of CCI rats. Our novel findings offer novel insights into the underlying mechanism through which EA effectively treats NP.

## 2 MATERIALS AND METHODS

### 2.1 Animals and CCI‐induced NP model

Male Sprague‐Dawley (SD) rats weighing 180 ~ 200 g were purchased from Shanghai SLAC Laboratory Animal Center (SCXK 2017–0005). All experimental rats were housed in the Animal Center of Shanghai Municipal Hospital of Traditional Chinese Medicine (TCM) at a room temperature of 22 ± 0.5°C, a 12–12 h light/dark cycle and were taken food and water freely. The animal study was approved by the Animal Experiments Ethical Committee of Shanghai Municipal Hospital of TCM (No. 021019) and followed the regulatory animal care guidelines of the National Institute of Health of the United States (Bethesda, MD, USA).

The CCI operation was established to induce NP modelling, as previous studies described.^19^, ^20^ Briefly, rats were deeply anesthetized with isoflurane, and the right sciatic nerve was exposed by making a skin incision and cutting through the connective tissue that separated the gluteus superficialis and biceps femoris muscles. Following this, four ligatures were meticulously applied to the sciatic nerve, with each one being spaced 1 mm apart. The extent of constriction was assessed by observing minor muscular quivering in the ipsilateral limb. Finally, the wound was closed with sutures in the muscle and staples in the skin. In the Sham group, similar procedures were performed on the right sciatic nerve without the application of any ligatures.

### 2.2 EA treatments

Following inhalation anaesthesia, the rats were immobilized and suspended by EA treatment as previously described.^21^, ^22^ Acupuncture needles were subsequently inserted into two acupoints: the Zusanli (ST36), located in the right hind limbs, and Baihui (GV20), located in the midline of the head. Subsequently, EA stimulation was administered using an SDZ‐II Electronic acupuncture instrument for a total of 30 min per day, with an intensity of 1 mA and a frequency of 2 Hz. Meanwhile, rats in the Sham group and CCI group underwent inhalation anaesthesia with isoflurane but did not receive any EA stimulation.

### 2.3 Drug administration

ML385 (Selleck, #S8790), an Nrf2 inhibitor, was purchased from Selleck (Houston, USA). Based on the results of preliminary experiments and previous studies,^23^ ML385 was dissolved in 100% DMSO to prepare a stock solution with a concentration of 100 mM and then diluted into phosphate buffer solution (PBS) (1:1000). The dosage of ML385 was applied via intrathecally injected in a volume of 10 μL daily into the L5–6 interspace. Rats of the control group were administered with vehicle (0.1% DMSO in PBS) only. The injections were conducted using 10 μL microinjector (Hamilton Company, Reno, NV).

### 2.4 Experimental design

**FIGURE 1:**
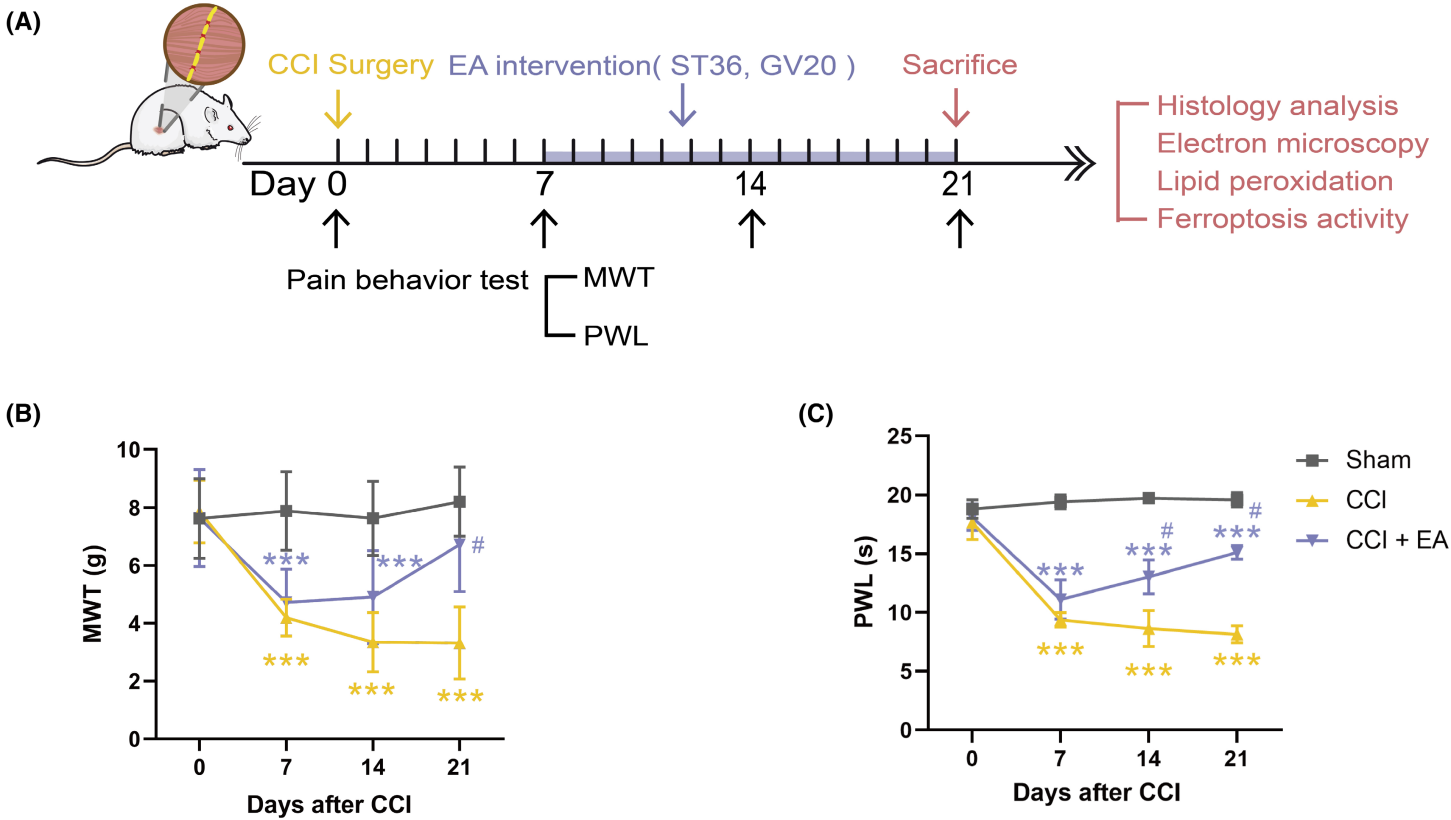
EA improved CCI‐induced NP phenotype. (A) The study protocol of EA treatment in CCI rats. (B) Effects of EA on MWT induced by CCI surgery. (C) Effects of EA on PWL in CCI‐induced NP models. *n* = 8–10. *p < 0.05, ***p < 0.001. vs. Sham group. ^#^ *p* < 0.05. vs. CCI group.

To explore the therapeutic role of EA on NP, 30 rats were randomly divided into three groups (*n* = 10 per group) in Experiment 1 (Figure 1): the Sham group, the CCI group (performed CCI operation) and the CCI + EA group (CCI rats treated EA). Seven days after CCI surgery, all rats in the EA group underwent EA stimulation for 14 consecutive days. Behavioural assessments were conducted at baseline and on Days 7, 14 and 21 post‐CCI surgery, followed by euthanasia of the rats and collection of spinal cord tissues for further analyses.

To investigate Nrf2 as a key EA therapeutic target for alleviating CCI‐induced pain hypersensitivity. In Experiment 2 (Figure 6), 25 rats were randomly divided into five groups: Sham, CCI, CCI + EA, CCI + ML385 and CCI + ML385 + EA group (*n* = 5 per group). For a period of 14 consecutive days, rats in CCI + ML385 group and CCI + ML385 + EA group were intrathecally injected with ML385 in a volume of 10 μL daily, while rats in the Sham, CCI and CCI + EA group received vehicle control (0.1%DMSO diluted by PBS) of 10 μL injections. In the CCI + ML385 + EA group, rats received ML385 microinjections 1 hour prior to EA treatment. Behavioural assessments were conducted at baseline and on Days 7, 14 and 21 post‐CCI surgery, followed by euthanasia of the rats and collection of spinal cord tissues for further analyses.

### 2.5 Behavioural tests

#### 2.5.1 Mechanical withdrawal threshold (MWT)

To detect the changes in mechanical hyperalgesia in rats, MWT was assessed using Von Frey fibres as previously described.^24^ Briefly, rats were housed in a 20 cm × 15 cm × 15 cm plexiglass observation chamber (with several compartments) with freedom of movement and accommodation for 30–60 min. When the rats were quiet, the Von Frey fibre was used to stimulate the paw between the right hind pads of the rat through the meshes of the metal frame. The experiment was repeated 3 times with an interval of 10 min, and the average value was taken as the measurement result.

#### 2.5.2 Paw withdrawal latency (PWL)

To assess nociceptive responses to thermal stimuli, PWL was determined by the radiant thermal apparatus (IITC, USA, II‐390). Briefly, the rats were placed in a plastic chamber to adapt for 30 min before testing. Once the rats stopped exploring and stood quietly, the radiant heat source was positioned under the glass floor directly beneath the mid‐plantar surface of the right hind paw. The intensity of the light source was set to 22% ~ 32% and the cut‐off time was 20 s, to avoid excessive heating injury. The latency time was recorded, and the evaluation was repeated 3 times, with an interval of 5–10 min each time, and the mean was calculated. All behavioural testing measurements were conducted by investigators in a blind manner.

### 2.6 Lipid peroxidation assay

The levels of malondialdehyde (MDA) and superoxide dismutase (SOD) in the L4‐L6 spinal cord of rats were assessed using the appropriate assay kits obtained from Solarbio (Beijing, China, BC0025 and BC0175) following the manufacturer's instructions.

### 2.7 Iron concentration assay

The concentration of iron in the L4‐L6 spinal cord of rats was measured utilizing an iron assay kit (Nanjing Jiancheng Bioengineering Institute, China, #A039‐2‐1) in strict accordance with the manufacturer's instructions. The absorbance of each well at a wavelength of 520 nm was measured using a microplate reader from BioTek.

### 2.8 Histology and Immunohistochemistry (IHC) staining

For histological staining, the L4‐L6 lumbar enlargement of the spinal cord was fixed in a 10% neutral buffered formalin solution. After 24 h, tissues were dehydrated and embedded in paraffin. Serial sections (4 μm thick) were cut and stained with haematoxylin–eosin (H&E) staining and Nissel staining solutions (Solarbio, G1430). The main observation location was the spinal dorsal horn on the lesioned side.

IHC staining protocol was performed as we previously described.^25^ Paraffin sections were rehydrated and digested in Proteinase K solution (Leagene, #H0310) for 20 min at 37°C, then treated with primary antibodies Nrf2 (Affinity, AF0639, 1:500) and Keap1 (Affinity, AF5266, 1:1000) overnight at 4°C, and then incubated with a Polink‐2 plus polymer HRP detection system kit (ZSGB‐BIO, #PV‐9001). The coloration was achieved using 3,3'‐Diaminobenzidine (DAB) solution. After counterstaining with haematoxylin, the slices were dehydrated, cleared and mounted. The stained sections were observed with a light microscope (Leica, DM6).

### 2.9 Immunofluorescence (IF) staining and co‐staining

The expression of NeuN (Abcam, ab279296, 1:1000) and ACSL4 (Abcam, ab155282, 1:100) in the spinal cord was determined by IF staining, as previously described.^26^ Moreover, to identify the type of cell undergoing ferroptosis, co‐staining of the biomarkers of microglia (Iba‐1), astrocytes (GFAP) or neurons (NeuN) with GPX4 were performed. Paraffin sections (4 μm thick) of the spinal cord were treated with primary antibodies anti‐rabbit GPX4 (Abcam, ab125066, 1:200) and anti‐mouse antibodies NeuN (Abcam, ab279296, 1:1000) or anti‐mouse Iba‐1 (Abcam, ab283319, 1:100) or anti‐chicken GFAP (Abcam, ab4674, 1:1000) overnight at 4°C, then incubated with fluorescent‐labelled secondary antibodies for 1 hour, and counterstained with DAPI. Finally, the sections were scanned using an Olympus VS120 whole slide imager.

### 2.10 Western blot

L4‐L6 enlargement of the spinal cord was harvested, and then, the protein was extracted with tissue lysate buffer (Beyotime). Protein contents were determined using a BCA protein assay kit (Beyotime). Equal amounts of protein (20 μg) were separated by SDS‐PAGE and transferred onto PVDF membranes (Millipore). Specific antibodies were used for incubation, and the proteins were visualized using the ChemiDOC western blot technique (BIO‐RAD). The following primary antibodies were used for western blot analysis: GPX4 (Abcam, ab125066, 1:1000), xCT (Abcam, ab175186, 1:1000), FTH1 (Abcam, ab183781, 1:1000), Nrf2 (Affinity, AF0639, 1:1000) and GAPDH (Beyotime, AF1186, 1:1000). GAPDH served as an internal control.

### 2.11 Transmission electron microscope (TEM)

The surgical extraction of the L5 spinal dorsal horn was followed by sectioning it into a 1 mm^3^ specimen, which was then immersed in glutaraldehyde. Then, the tissue specimen was fixed with 1% Russian acid, followed by ethanol dehydration, acetone extraction and embedding procedures. The resulting samples were sliced into 70 nm thick and stained with 2% uranyl acetate solution for 15 min and a lead citrate solution for 5–10 min. The ultrastructure of mitochondria was observed under a Hitachi TEM system. Ten sections from each specimen were selected for analysis. The key reagents in this study are shown in Table 1.

**TABLE 1:** Key resources.

Regent or Resource	Source	Identifier
Antibodies		
xCT	Abcam	ab175186
GPX4	Abcam	ab125066
FTH1	Abcam	ab183781
GAPDH	Beyotime	AF1186
Nrf2	Affinity	AF0639
Keap1	Affinity	AF5266
NeuN	Abcam	ab279296
ACSL4	Abcam	ab155282
Iba‐1	Abcam	ab283319
GFAP	Abcam	ab4674
Critical Commercial Assays		
Malondialdehyde (MDA) Content Assay Kit	Solarbio	BC0025
Superoxide Dismutase(SOD) Activity Assay Kit	Solarbio	BC0175
Tissue Iron Assay Kit	Nanjing Jiancheng Bioengineering Institute	A039‐2‐1

### 2.12 Statistical analysis

Data were presented as mean ± standard deviation (SD). The statistical analysis was performed using the SPSS 21.0 statistical software. Repeated measures analysis of variance was used to compare differences in behavioural tests. One‐way analysis of variance (*ANOVA*) and two‐way ANOVA followed by least significant difference (*LSD*) were used to compare differences among multiple groups. *p* value <0.05 was considered statistically significant.

## 3 RESULTS

### 3.1 EA improves pain hypersensitivity in CCI‐induced NP rats

To evaluate the analgesic effects of EA on NP disorder, CCI rats received EA stimulation at Zusanli (ST36) and Baihui (GV20) acupoints for 14 consecutive days after 7 days of NP modelling. The pain responses, measured as MWT and PWL, were evaluated before CCI surgery and on Days 7, 14 and 21 after CCI operation (Figure 1A). Compared to the Sham group, CCI rats exhibited significant decreases in MWT and PWL. In contrast, 14 days of EA treatment showed substantial improvements in MWT and PWL in NP rats, restoring them to 81.9% and 77.2% of the Sham group, respectively (Figure 1B,C). These results suggest that EA could obviously attenuate CCI‐induced pain sensitization in the NP model, and its analgesic effect intensifies over time.

### 3.2 EA protects neuronal damage and loss in the spinal cord of CCI‐induced NP rats

**FIGURE 2:**
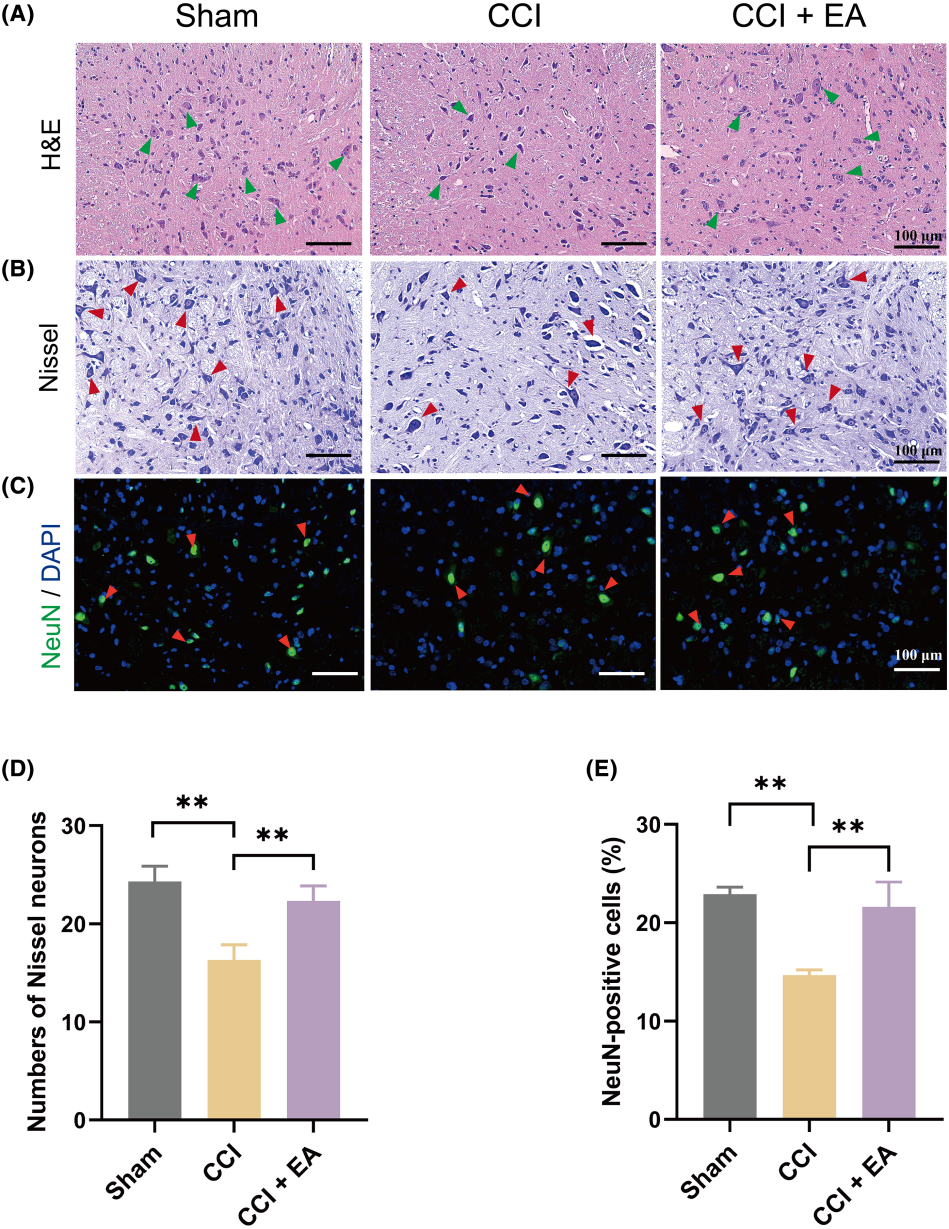
EA protected the neuron damage and loss in the spinal dorsal horn caused by NP rats. (A) H&E staining (spinal cord dorsal horn) of each group. Scale bar, 100 μm. Green arrows pointed out the neurons. (B) Nissel staining in the spinal cord dorsal horn among groups. Red arrows pointed out the Nissel bodies. Scale bar, 100 μm. (C) IF staining of NeuN (red arrows) in the spinal cord dorsal horn among groups. Scale bar, 100 μm. (D) The representation of the number of Nissl‐stained cells in the spinal dorsal horn among groups. (E) Quantification of NeuN‐positive cells among groups. Data presented as means ± s.d. **p < 0.01. *n* = 3.

To evaluate the impact of EA treatment on neuronal damage in the spinal cord of CCI‐treated rats, the morphologic changes of the spinal cord were assessed using H&E staining. Compared to the Sham group, CCI rats exhibited disorganized neurons in the spinal dorsal horn, indicative of neuron damage following the CCI operation. On the contrary, EA treatment partially inhibited the structural alteration in the spinal cord neurons in NP rats (Figure 2A). To further investigate the function of neurons, Nissl staining was performed. Consistently, Nissel staining results showed a lower number of Nissl substances (bodies) in the CCI group compared to the Sham rats. Additionally, some remaining neurons displayed enlargement of Nissl substance and apparent nuclear envelope thickening, indicating neuronal damage and loss in the NP model. Gratifyingly, EA therapy could restore the reduction in Nissel bodies (Figure 2B,D).

To confirm the neuroprotective effect of EA against NP, we further detected the changes in neuronal marker expression (NeuN). In the spinal dorsal horn of CCI rats, the IF results revealed that the expression of NeuN was remarkably reduced to 36.0% compared to the Sham group, while treatment with EA increased the proportion of NeuN‐positive cells to 17.1% compared to the CCI rats without EA treatment (Figure 2C,E). These data demonstrate that EA treatment effectively attenuates the neuron damage and loss in the spinal dorsal horn of CCI rats.

### 3.3 EA attenuates the ferroptosis phenotype in CCI‐induced NP rats

**FIGURE 3:**
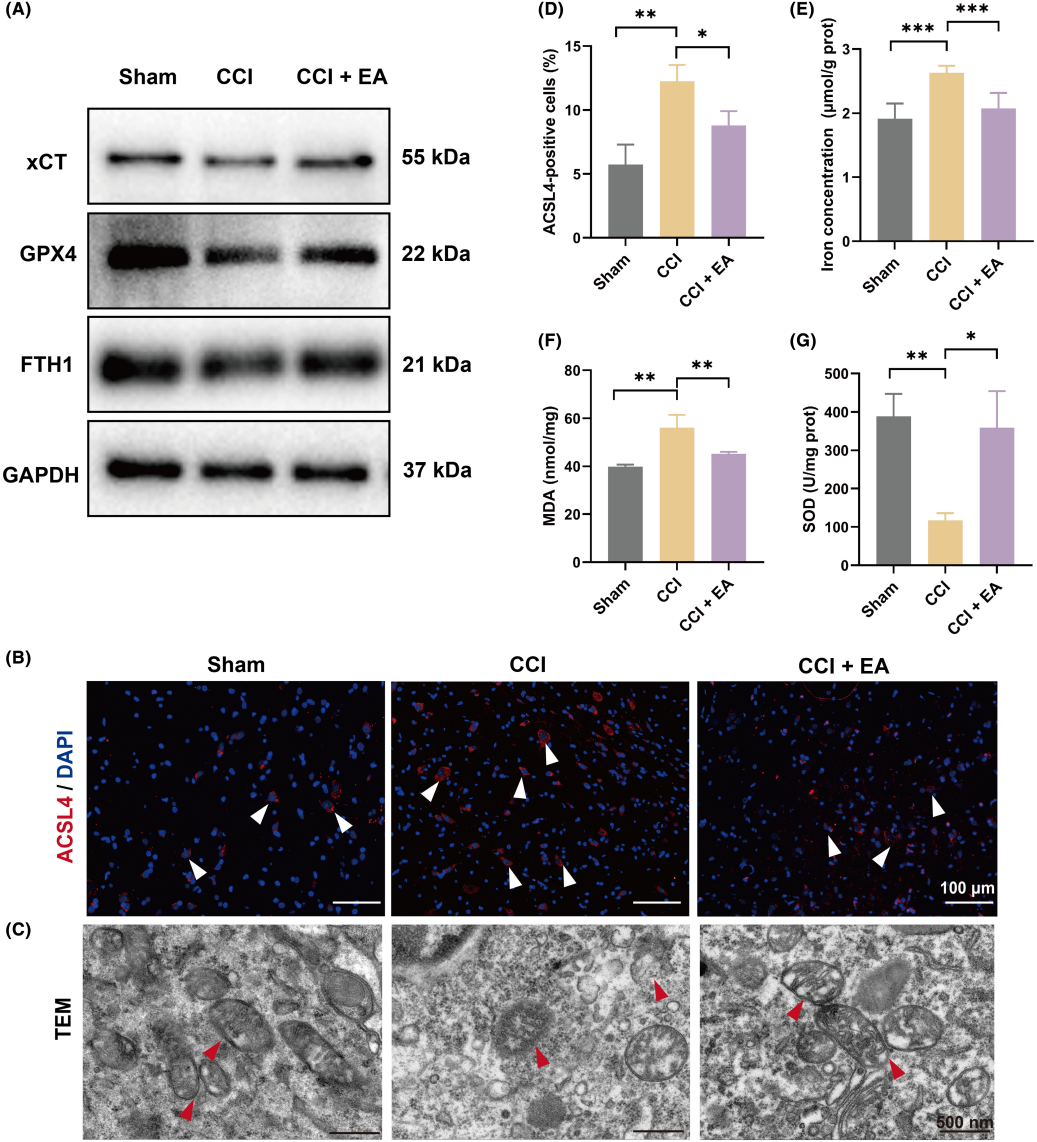
EA attenuated CCI‐induced ferroptosis phenotype in the spinal cord. (A) The protein expression of xCT, GPX4 and FTH1 in the spinal cord of each group. GAPDH was used as an internal control. *n* = 3. (B) IF staining of ACSL4 in the dorsal horn of the spinal cord of each group. Scale bar, 100 μm. *n* = 3. (C) Changes of the mitochondrial ultrastructure (red arrows) in the spinal cord dorsal horn among groups by TEM. Scale bar, 500 nm. *n* = 3. (D) Quantitative analysis of ACSL4‐positive cells in each group. *n* = 3. (E) Changes in iron levels in each group. *n* = 5–6. (F) The level of MDA in the spinal cord of each group. *n* = 4–7. (G) The level of SOD in the spinal cord of each group. *n* = 3–4. Data presented as means ± s.d. *p < 0.05, **p < 0.01, ***p < 0.001.

To gain a better understanding of the regulation of ferroptosis by EA in the NP model, we detected the alterations in ferroptosis‐related biological characteristics in CCI‐induced NP rats. Firstly, the levels of key anti‐ferroptosis protein, xCT and GPX4, as well as critical iron storage protein, FTH1, were detected by western blot. The results showed significant downregulation of xCT, GPX4 and FTH1 in the spinal cord of CCI rats, while EA treatment increased the expression of these proteins (Figure 3A). Furthermore, IHC staining analysis of a critical pro‐ferroptosis marker, ACSL4, revealed overexpression in CCI‐treated rats, which was markedly reduced by EA treatment in NP rats (Figure 3B,D). In addition, the microstructure changes of mitochondria were observed by TEM. As expected, Sham rats displayed long, interconnected mitochondria with intact outer membranes and well‐developed cristae, and CCI surgery induced plenty of mitochondrial damage, characterized by reduced or absent cristae. In the EA group, a partial recovery of the damaged mitochondrial cristae was observed (Figure 3C). These findings indicate that EA effectively counteracts oxidative stress and reduces ferroptosis activity in the spinal cord of NP rats.

Furthermore, we detected the changes in iron concentration and lipid peroxidation metabolites in the spinal cord. The result showed a significant increase in iron levels in NP rats compared to the Sham group, whereas EA significantly decreased iron contents (Figure 3E), suggesting that EA could inhibit aberrant iron accumulation in the spine of NP rats. Subsequently, we detected the major antioxidant product, SOD and lipid peroxidation‐related indicator, MDA, in the spinal cord. We found that CCI surgery induced a decrease in SOD and an increase in end products of lipid peroxidation, MDA, both associated with disordered expressions due to CCI surgery, but restored by EA administration (Figure 3F,G). The above results indicate that EA treatment could attenuate the CCI‐induced ferroptosis phenotype in the spinal cord of NP rats.

### 3.4 EA inhibits neuronal ferroptosis in the spinal cord of CCI‐induced NP rats

**FIGURE 4:**
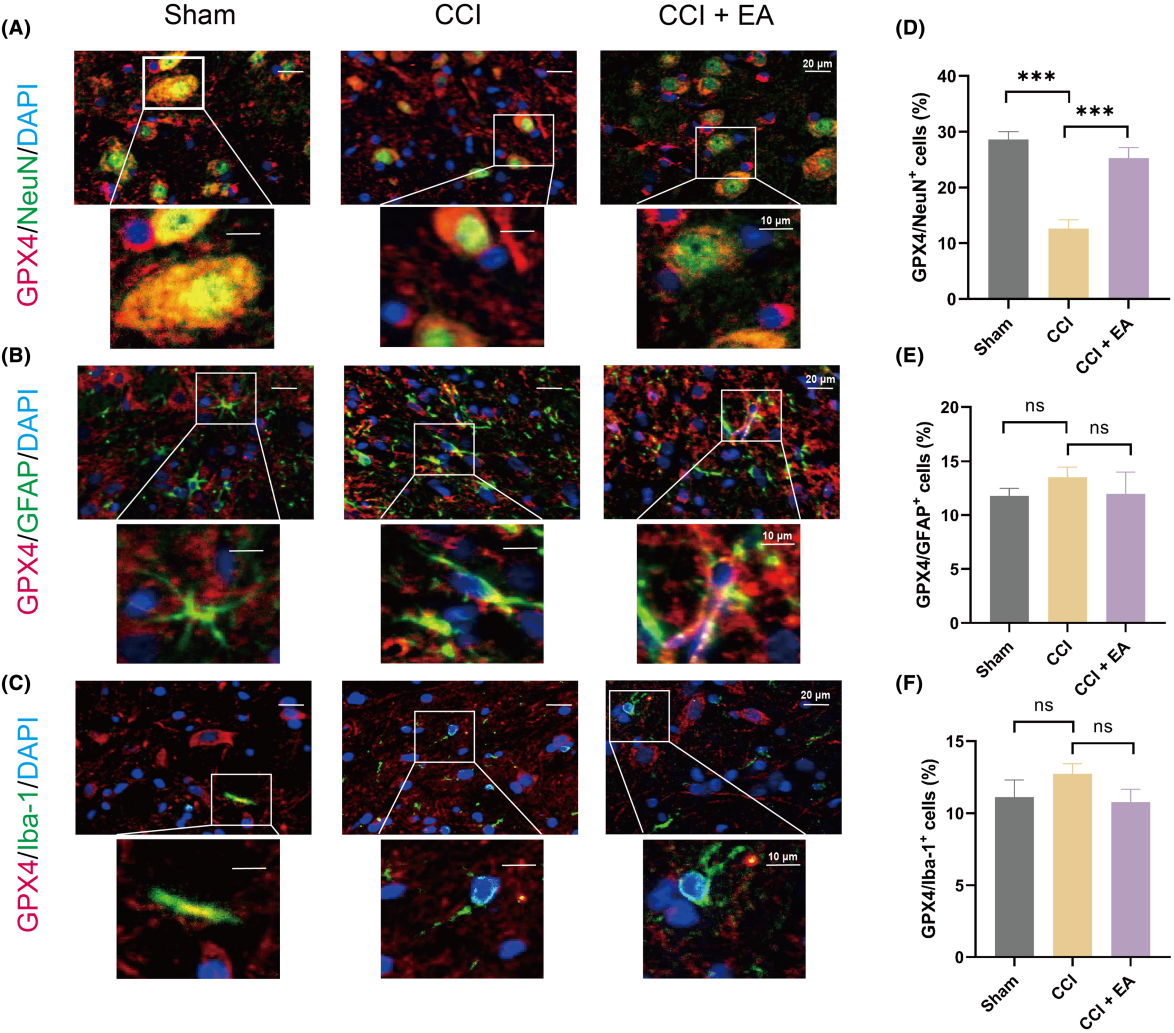
EA restored the GPX4‐mediated ferroptosis in neuron cells but not in microglia or astrocytes following CCI operation. (A) Representative images of immunofluorescent analysis of NeuN^+^ (green), GPX4^+^(red) maker and DAPI (blue) staining of nuclei in the spinal cord dorsal horn among groups. Scale bars, 20 μm (top), 10 μm (bottom). (B) Representative images of immunofluorescent analysis of GFAP^+^(green), GPX4^+^(red) maker and DAPI (blue) staining of nuclei in the spinal cord dorsal horn among groups. Scale bars, 20 μm (top), 10 μm (bottom). (C) Representative images of immunofluorescent analysis of Iba‐1^+^ (green), GPX4^+^(red) maker and DAPI (blue) staining of nuclei in the spinal cord dorsal horn among groups. Scale bars, 20 μm (top), 10 μm (bottom). Quantification analysis the number of GPX4/NeuN^+^ (D), GPX4/GFAP^+^ (E) and GPX4/ Iba‐1^+^(F) in the spinal cord dorsal horn. Data presented as means ± s.d. ***p < 0.001, *n* = 3.

Moreover, to illustrate the specific cellular targets of EA in the regulation of ferroptosis in the spinal cord during NP modelling, we detected the co‐expression of GPX4 with cellular biomarkers of astrocytes (GFAP), microglia (Iba‐1) and neurons (NeuN). Interestingly, EA remarkably increased the double immunostaining of GPX4/NeuN (Figure 4A) and quantitative analysis revealed an approximate 1.3‐fold upregulation of GPX4 expression in neurons within the ipsilateral spinal cord of the EA group compared to the CCI group (Figure 4D). However, EA therapy failed to stimulate the co‐expression of GPX4/Iba‐1 or GPX4/GFAP (Figure 4B,C,E,F). Collectively, treatment with EA specifically inhibits GPX4‐mediated neuronal ferroptosis, while exerting no impact on microglia or astrocytes.

### 3.5 EA enhances Nrf2 expression and inhibits the Keap1 expression in the spinal cord

**FIGURE 5:**
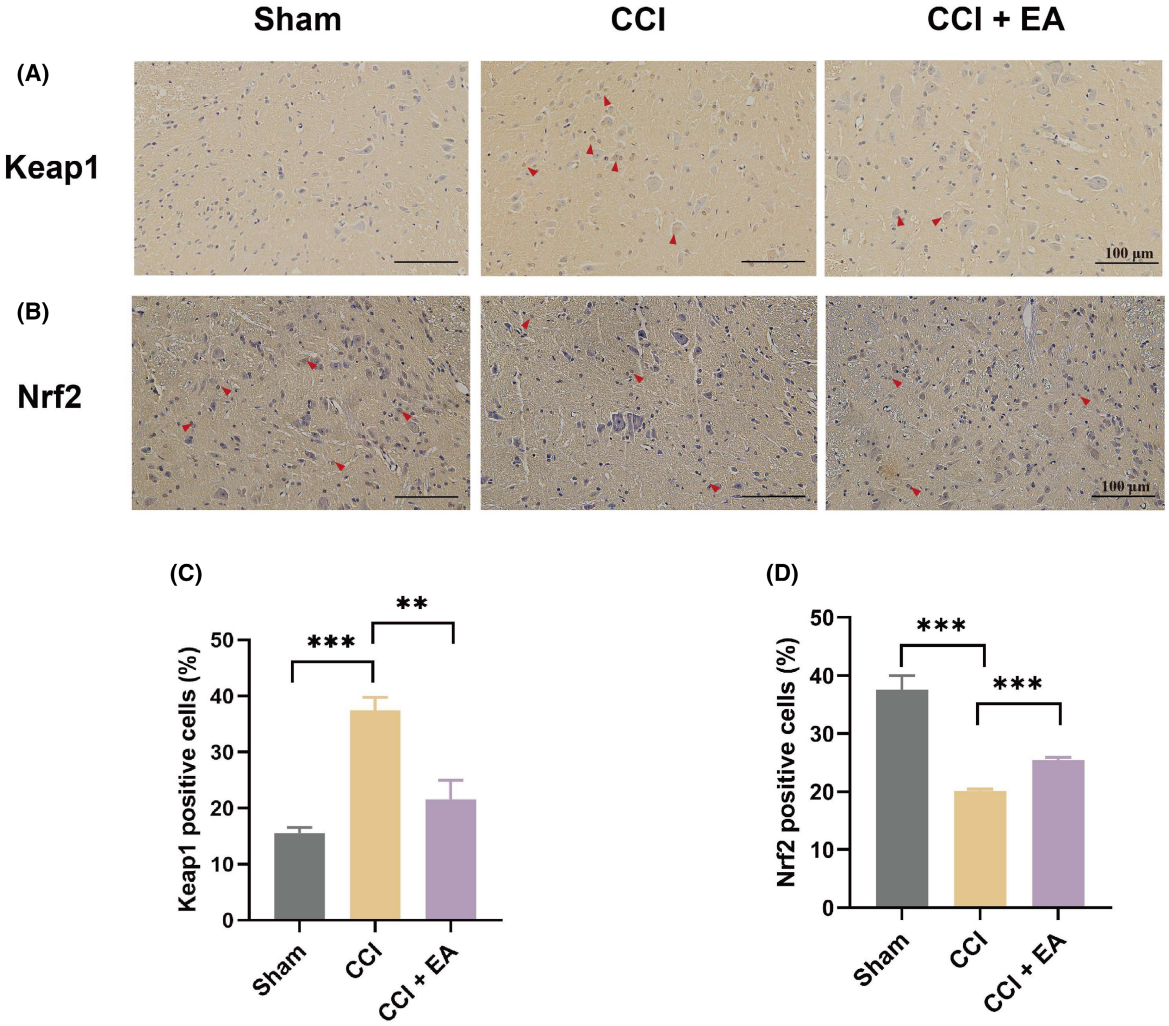
EA increased the activity of Nrf2 and a concurrent reduction in Keap1 expression in the spinal cord of CCI rats. (A) IHC staining of Keap1 in the spinal cord dorsal horn of each group. Scale bar, 100 μm. (B) IHC staining of Nrf2 in the spinal cord dorsal horn of each group. Scale bar, 100  μm. (C) Quantitative analysis of Keap1‐positive cells in the spinal cord dorsal horn. (D) Quantification analysis of the number of Nrf2‐positive cells in the spinal cord dorsal horn. Data presented as means ± s.d. **p < 0.01, ***p < 0.001. *n* = 3.

The Nrf2 signalling pathway has been identified as a crucial regulator of ferroptosis in peripheral nerve injuries and brain trauma.^27^, ^28^ Nrf2 is normally restricted to the cytoplasm as part of the Keap1‐Nrf2 complex.^29^ However, in response to oxidative stress, the Keap1–Nrf2 complex dissociates, authorizing Nrf2 to translocate into the nucleus, where it binds antioxidant response elements and regulates the transcription of downstream target genes, such as xCT, FTH1 and GPX4.^30^, ^31^, ^32^ To observe the effect of EA on Nrf2 and Keap1 regulation, we used the IHC assay to detect the expression of Nrf2 and Keap1 in spinal cord. In the spinal cord of CCI rats, the expression level of Nrf2 exhibited a reduction of approximately 50%, whereas the expression of Keap‐1 demonstrated an increase of more than twofold. However, upon EA treatment, these effects were effectively reversed, leading to a notable 26.3% increase in Nrf2 expression and a significant 42.4% decrease in Keap1 expression compared to the CCI group (Figure 5A–D). Consequently, it can be inferred that EA treatment promoted the Nrf2 expression and inhibited the Keap1 expression, and EA may have the potential to attenuate the extent of ferroptosis by activating Nrf2 signalling.

### 3.6 Suppression of Nrf2 antagonizes EA's protective effect against neuronal ferroptosis in NP model rats while marginally diminishing its analgesic effect

**FIGURE 6:**
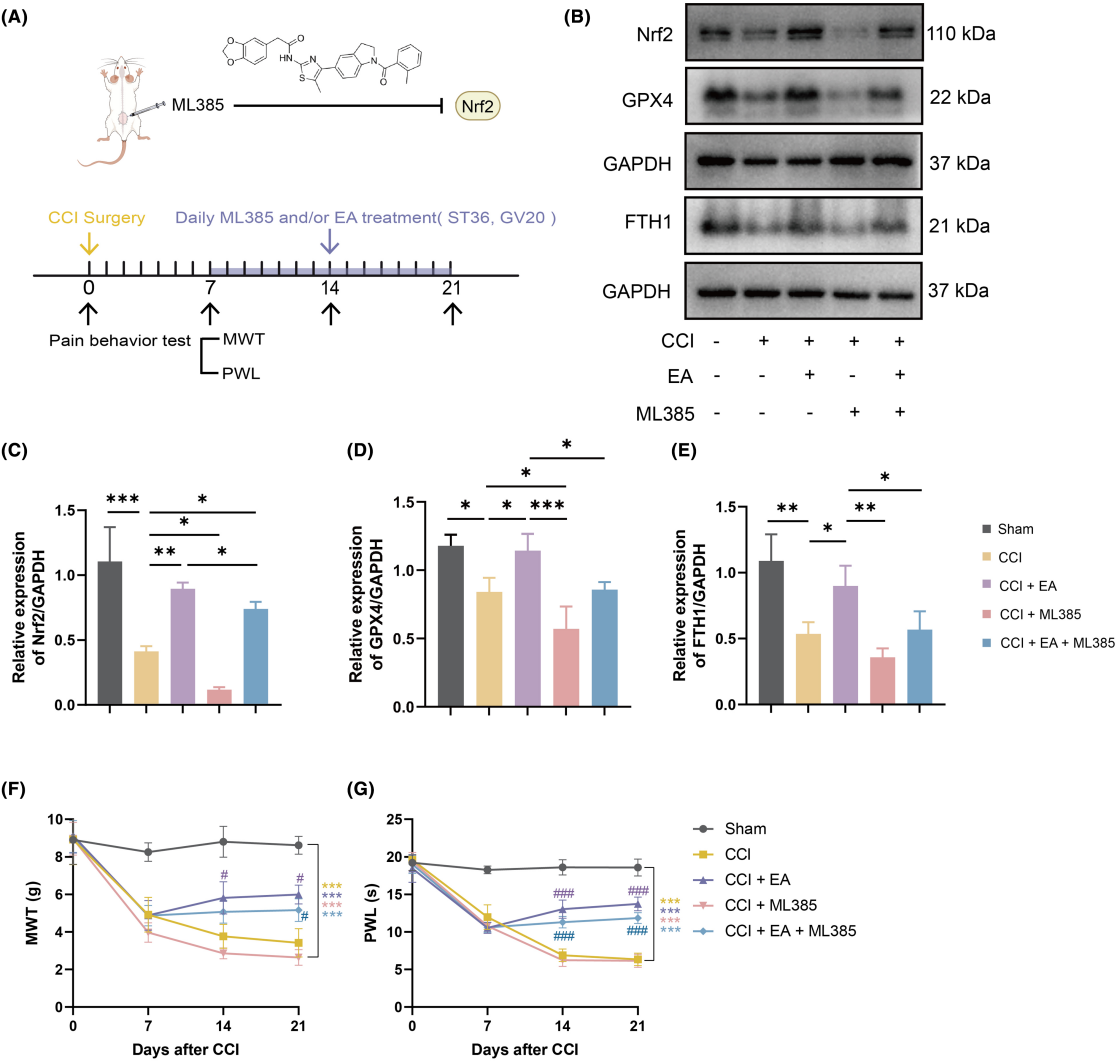
EA inhibited ferroptosis by partial activation of Nrf2 signalling, but inhibition of Nrf2 did not abolish the analgesic effect of EA therapy. (A) Schematic representation of the Nrf2 inhibitor ML385 upon intrathecal injection into the spinal cord. The study protocol of EA treatment in CCI rats following the use of ML385. (B) The protein expression of Nrf2, GPX4 and FTH1 in the spinal cord of each group. GAPDH was used as an internal control. (C–E) The relative expression of Nrf2, GPX4 and FTH1 protein in each group. Data presented as means ± s.d. *p < 0.05, **p < 0.01, ***p < 0.001. *n* = 3. The effects of EA on MWT (F) and PWL (G) in CCI‐induced NP models. *n* = 5. Data presented as means ± s.d. *p < 0.05, ***p < 0.001. vs. Sham group. ^#^ *p* < 0.05, ^###^ *p* < 0.001. vs. CCI group.

Subsequently, to further ascertain the role of Nrf2 signalling on the protective effects of EA on neuronal ferroptosis and NP progression, NP model rats were treated with ML385 (an Nrf2 inhibitor) alongside EA intervention 7 days after CCI surgery (as depicted in Figure 6A). Notably, ML385 administration further reduced the expression of Nrf2 and its downstream ferroptosis‐related proteins, GPX4 and FTH1, in the spinal cord, compared to NP rats. Furthermore, the combined intervention of ML385 with EA significantly reversed EA‐induced upregulation of Nrf2, GPX4 and FTH1, compared to NP rats treated EA alone (Figure 6B–E). Intriguingly, behavioural analysis results indicated that, when compared to the NP model group, administration of ML385 resulted in a non‐significant decrease in MWT in NP model rats, while no significant difference was observed in PWL. Additionally, the combined intervention of ML385 with EA led to a non‐significant decrease in both MWT and PWL in NP model rats, compared to the EA treated rats (Figure 6F,G).

## 4 DISCUSSION

Increasing studies have demonstrated the therapeutic benefits of EA in treating NP and its underlying molecular mechanisms are being actively investigated.^7^, ^33^, ^34^ In this study, we found that EA effectively ameliorated mechanical and thermal hypersensitivities induced by CCI surgery. Moreover, EA exhibited inhibitory effects on the neuronal ferroptosis phenotype in the spinal cord of CCI rats, as evidenced by the upregulation of xCT, GPX4 and FTH1 expression, downregulation of ACSL4 expression, restoration of iron overload and normalization of dysregulated SOD and MDA levels. Meanwhile, EA alleviated the aberrant mitochondrial morphology. Furthermore, EA treatment activated Nrf2 signalling by upregulating reduced Nrf2 and decreasing elevated Keap1 expression in the spinal of NP rats, and suppression of Nrf2 signalling using ML385 significantly blocked EA's therapeutic effect on neuronal ferroptosis activity. These findings suggest that EA treatment at acupoints ST36 and GV20 may protect against NP by inhibiting GPX4‐mediated neuronal ferroptosis in the spinal cord, partly through the activation of Nrf2 signalling.

Several studies have proven the significant neuroprotective effect of the widely used acupoints ST36 and GV20 in the management of neurological diseases.^35^, ^36^, ^37^ Specifically, the ST36 has proven to be beneficial in alleviating chronic pain.^38^ Consistent with previous findings, we found that EA stimulation acupoints at ST36 and GV20 markedly decreased pain hypersensitization induced by CCI surgery, with the anti‐nociceptive effect increased in a time‐dependent manner. Existing research suggests that the persistent analgesic effect of EA on NP may be attributed to the improvement in neuronal activities within the spinal dorsal horn of chronic neuralgia rats.^7^, ^39^ Consistently, our data revealed that EA treatment effectively increased specific neuronal marker NeuN expression in the spinal cord, leading to reduced neuron damage and loss is one of the mechanisms of the neuroprotective effect of EA treatment.

Increasing evidence indicates that ferroptosis is an important pathophysiological mechanism of several neuronal injury diseases such as ischemic stroke,^40^ traumatic brain injury.^41^ Emerging research has confirmed the close association between ferroptosis and NP development, with the inhibition of ferroptosis contributing to the amelioration of pain hypersensitivities in CCI rats.^15^, ^16^ The ferroptosis process involves impairments in xCT function that trigger ferroptosis by reducing glutathione synthesis and inactivating GPX4, followed by the oxidation of intracellular lipids through a Fenton‐like chemical reaction, resulting in the generation of metabolites (e.g., MDA).^42^ ACSL4 influences ferroptosis sensitivity by shaping cellular lipid composition, as its overexpression correlates with increased susceptibility to ferroptosis induction, while its inhibition prevents ferroptosis.^43^ Consistent with the existing literature, our findings indicate that CCI surgery led to declined xCT and GPX4 levels, accompanied by increased ACSL4. Nevertheless, EA therapy efficiently rectified aberrant xCT and GPX4 expression, while decreasing ACSL4 expression. MDA is a biomarker for lipid peroxidation and oxidative protein damage, while SOD and GPX4 are endogenous antioxidant factors for maintaining the homeostasis of the cellular redox environment.^14^ Our results found EA treatment significantly reduced MDA levels and increased SOD and GPX4 activity, suggesting a substantial reduction in lipid peroxidation within the spinal cord.

The previous study has highlighted that excessive accumulation of lipid peroxidation products heavily relies on dysregulated iron levels.^10^ FTH1 is recognized as the major iron storage protein and plays a crucial role in the ferroptosis process.^44^ Decreased FTH1 expression can contribute to the onset of ferroptosis.^45^ In this study, we also found administration of EA improved the spinal iron deposition and elevated FTH1 protein expression in NP rats, which led us to speculate that modulation of iron metabolism may represent one of the mechanisms underlying EA treatment.

In addition, *Gpx4* is not only required for the survival of neurons but also for maintaining mitochondrial function. Overexpression of *Gpx4* has been shown to protect neurons and mitochondrial structure against oxidative injury,^46^, ^47^ while *Gpx4* ablation significantly reduces the number of NeuN‐positive cells in the hippocampus and triggers neuronal ferroptosis,^48^, ^49^ indicating that GPX4 serves as a key regulator of neuronal ferroptosis death and neuronal fate. Our study revealed that EA not only upregulated GPX4 protein expression in the spinal dorsal horn but also increased GPX4 expression in the neuron cells, rather than microglia and astrocytes, indicating that the predominant effect of EA in targeting ferroptosis occurs in neuron cells.

Furthermore, the previous study has reported the beneficial role of Nrf2 signalling in treating NP,^50^ while emerging evidence also has shown activation of Nrf2 signalling by promoting the transfer of Nrf2 to the nucleus led to the upregulation of GPX4 and FTH1, thus modulating ferroptosis in various bioprocesses.^51^ In the present study, we found that EA treatment could upregulate the expression of Nrf2 while downregulating the expression of Keap1 in the CCI‐induced NP rats. Furthermore, inhibition of Nrf2 signalling using ML385 antagonized the protective effect of EA against neuronal ferroptosis by reversing EA‐induced upregulation of Nrf2, GPX4 and FTH1 in NP model rats. Intriguingly, inhibition of Nrf2 signalling resulted in a non‐significant aggravation in the CCI‐triggered pain hypersensitivities, which was consistent with the previous study.^23^ Also, Nrf2 inhibitor ML385 could not prevent EA from improving NP behavioural outcomes. Notably, EA alleviates NP via multiple signalling pathways, such as the JAK/STAT,^52^ AMPK/mTOR^53^ and BDNF/TrκB signalling pathway.^54^ Nrf2 signalling may be indirectly involved in the amelioration of NP. Thus, the above findings lead us to speculate that EA treatment relieves NP through multiple mechanisms, except the activation of Nrf2 signalling.

Finally, our study does possess some limitations. We solely described the analgesic effect of EA mediated by regulating oxidative stress and the ferroptosis‐specific indicators in NP rats. However, the mechanism of how EA treatment on ST36 and GV20 remotely inhibits neuronal ferroptosis activity remains unclear. Future studies should employ ferroptosis inducers such as Erastin or RSL3 (GPX4 inhibitor) to elucidate the potential molecular mechanisms through which EA counteracts spinal ferroptosis. Moreover, based on current data, the role of EA in mediating pain relief via other central regions (e.g., brain) or peripheral nerves (e.g., dorsal root ganglion) remains inconclusive. To better understand EA's analgesic effect, we will in the future explore if EA can regulate ferroptosis in the hippocampus or dorsal root ganglion.

## 5 CONCLUSION

**FIGURE 7:**
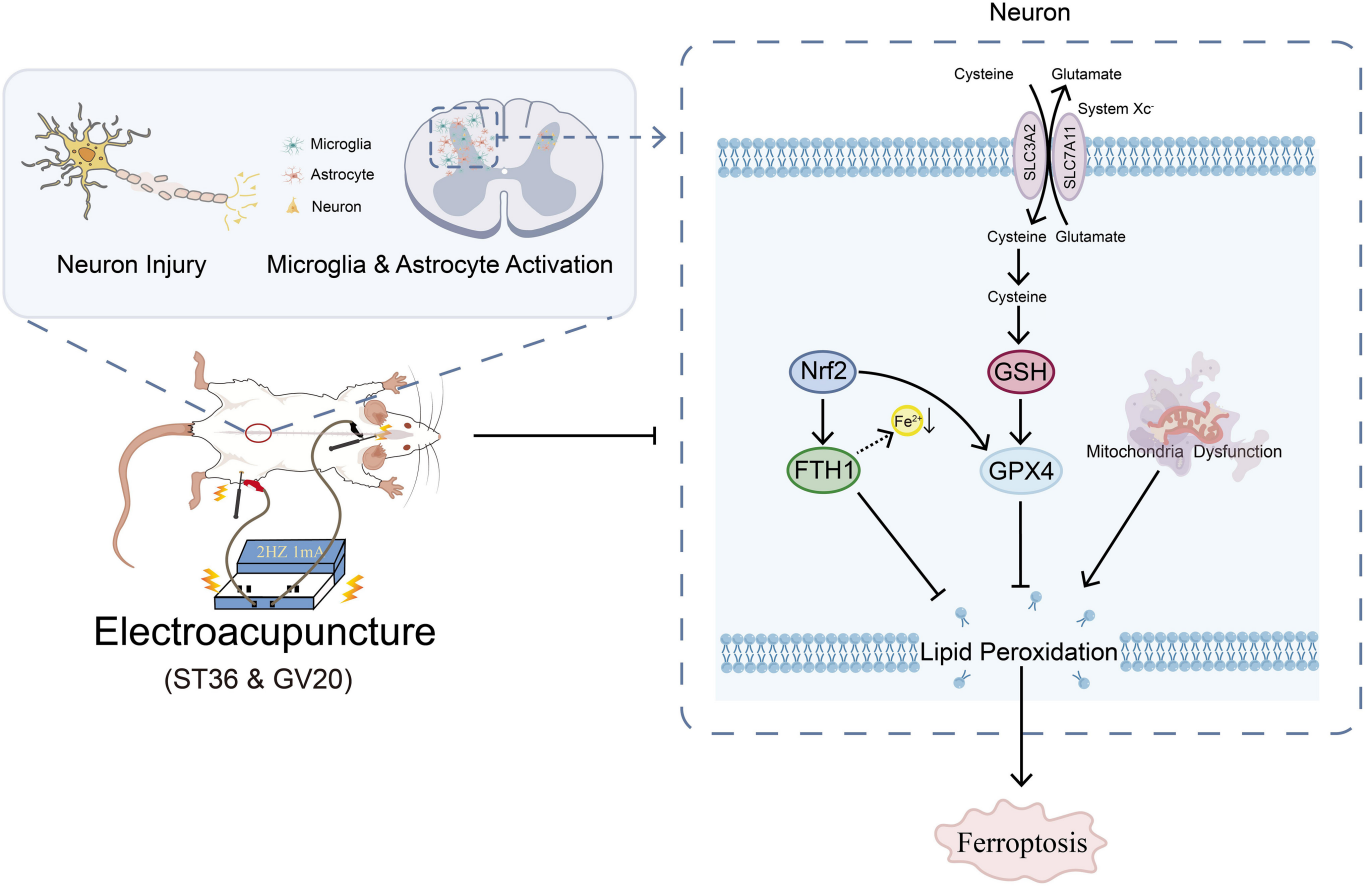
A schematic diagram illustrating the mechanism of EA treatment in peripheral nerve injury‐induced NP.

This study demonstrates the remarkable efficacy of EA intervention in ameliorating pain hypersensitivities induced by CCI and protecting neurons against injury and loss in NP rats. Moreover, EA effectively reverses GPX4‐mediated neuronal ferroptosis in NP rats. Furthermore, we provided evidence that EA may promote the expression of Nrf2 and suppress Keap1 activity in the spinal cord, and blockade of the Nrf2 pathway partially inhibited the effectiveness of EA against ferroptosis (Figure 7). These findings provide the first in vivo evidence highlighting EA therapy's potential to protect against NP by suppressing neuronal ferroptosis in the spinal cord. These findings unveil novel prospects for the prevention of this particular form of cell death in the context of NP.

## AUTHOR CONTRIBUTIONS

**Chunchun Xue:** Data curation (lead); funding acquisition (supporting); investigation (lead); writing – original draft (lead). **Wenyun Kui:** Data curation (lead); formal analysis (lead); investigation (lead); methodology (lead). **Aiping Huang:** Data curation (lead); formal analysis (supporting); funding acquisition (lead). **Yanan Li:** Data curation (equal); formal analysis (equal); methodology (equal). **Lingxing Li:** Data curation (equal); formal analysis (equal). **Zhen Gu:** Data curation (equal); formal analysis (equal). **Lei Xie:** Data curation (equal); formal analysis (equal). **Shuyi Kong:** Data curation (equal); formal analysis (equal). **Jun Yu:** Data curation (equal); formal analysis (equal). **hongfeng Ruan:** Conceptualization (lead); methodology (equal); supervision (lead); writing – review and editing (supporting). **Kaiqiang Wang:** Conceptualization (lead); funding acquisition (lead); writing – review and editing (lead).

## FUNDING INFORMATION

This work was funded by the following grants: Shanghai Science and Technology Planning Project (No. 20Y21903100), Shanghai Three‐year Action Plan for Further Accelerating the Development of Traditional Chinese Medicine [No. ZY(2018–2020)‐ZYBZ‐06], Budget Project of Shanghai University of TCM (No.2020LK063), Shanghai University of TCM Excellent Talents Training Program (No.TCM[2020]10), Shanghai University of TCM Xinglin Young Talent Training System‐Xinglin Scholars Project (No.TCM[2020]23), Traditional Chinese Medicine Science and Technology Development Project of Shanghai Medical Innovation & Development Foundation (No.WL‐HBMS‐2022002 K, WL‐HBMS‐2022003 K) and partly supported by Clinical Research Plan of Shanghai Hospital Development Center (No.SHDC2020CR3102B).

## CONFLICT OF INTEREST STATEMENT

The authors declare that there are no conflicts of interest.

## Data Availability

The original contributions presented in the study are included in the article/supplementary materials, and further inquiries can be directed to the corresponding authors.
